# Prevalence and predictors of polypharmacy prescription among type 2 diabetes patients at a tertiary care department in Ningbo, China: A retrospective database study

**DOI:** 10.1371/journal.pone.0220047

**Published:** 2019-07-17

**Authors:** Jialin Li, Kaushik Chattopadhyay, Miao Xu, Yanshu Chen, Fangfang Hu, Xingzhen Wang, Li Li

**Affiliations:** 1 Department of Endocrinology and Metabolism, Ningbo First Hospital, Ningbo, Zhejiang Province, PR China; 2 Division of Epidemiology and Public Health, School of Medicine, The University of Nottingham, Nottingham, United Kingdom; Medical University Graz, AUSTRIA

## Abstract

**Objectives:**

To determine the prevalence of polypharmacy prescription among type 2 diabetes (T2DM) patients at a tertiary care department in Ningbo, China, and to determine factors that independently predict this polypharmacy prescription.

**Methods:**

A retrospective cross-sectional study was conducted using an existing computerised medical records database. This database was screened from 2012 to 2017 for adult patients with T2DM and parameters like prescribed medicines and socio-demographic, behavioural and other medical information. Polypharmacy prescription was defined as the simultaneous prescription of ≥5 medicines by the clinician at the time of discharge for daily usage by the patient as part of his/her long-term treatment plan.

**Results:**

The study inclusion criteria were satisfied by 3370 T2DM patients. Over a 5-year period, 72.2% (n = 2432) of T2DM patients were prescribed polypharmacy. On an average, eight medicines were prescribed to them. The odds of polypharmacy prescription increased with patients’ age (18–39 years: 1; 40–59 years: OR 1.86, 95% CI 1.28–2.71; and ≥60 years: 2.42, 1.65–3.55), duration of T2DM (≤1 year: 1; >5–10 years: 1.70, 1.10–2.62; and >10 years: 2.55, 1.68–3.89), and length of hospital stay (≤5 days: 1; >5–10 days: 2.43, 1.86–3.17; and >10 days: 2.99, 2.24–3.99), and were higher in those with poor blood glucose level (2.09, 1.67–2.62) and with comorbidities like other endocrine, nutritional and metabolic diseases (2.24, 1.76–2.85), circulatory system diseases (4.35, 3.62–5.23), skin and subcutaneous tissue diseases (1.64, 1.04–2.59), and musculoskeletal system and connective tissue diseases (1.61, 1.27–2.03). The odds of polypharmacy prescription were lower in those with comorbidities like neoplasms (0.51, 0.36–0.70) and during pregnancy, childbirth and the puerperium (0.06, 0.01–0.49).

**Conclusions:**

Around three fourth of T2DM patients at the tertiary care department were prescribed polypharmacy, and the predictors were identified. The study findings could be taken into consideration in future interventional studies aimed at supporting medicines optimisation (and deprescribing) among these patients.

## Introduction

Type 2 diabetes (T2DM), a complex metabolic disorder, has major health, social and economic consequences. Globally, China has the largest T2DM epidemic. Currently, around 114 million (11%) adults are living with T2DM, which is expected to rise to 120 million by 2045 [1].

Ningbo, an economically developed city, is located in the northeast Zhejiang province of China. In 2015, the T2DM prevalence in adults over 40 years of age in Ningbo was 21% [2]. At a tertiary care department (Department of Endocrinology and Metabolism, Ningbo First Hospital), more than 50% of T2DM patients receiving treatment had poor glycaemic control and vascular complications [3]. The study was conducted at Ningbo First Hospital. This is a tertiary care hospital (with 1600 beds), which is primarily responsible for delivering specialist health services and for performing a larger role in medical education and research [4,5]. Nationals (including T2DM patients) can visit any Chinese hospital of their choice and is not based on a referral system by the general practitioners. Thus, this hospital is visited by local people, as well as those from surrounding areas [4].

The concurrent prescription or usage of multiple medicines is known as polypharmacy [6]. Polypharmacy can lead to interactions between drugs and results in adverse drug events [7–9]. It can have a negative effect on the T2DM patient, their family/carer, the health system and the economy. In patients, it can adversely affect their adherence to medicines, quality of life and life expectancy, can lead to suboptimal blood glucose control and hospital admissions, and can increase severe hypoglycaemia risk and healthcare costs [10–14]. Studies conducted in different diabetes (including T2DM) populations and settings have reported a range of polypharmacy prevalence figures (6–85%) [15–24].

Until now, no research has been conducted to explore polypharmacy prescription among T2DM patients at this tertiary care department. The study objectives were to determine the prevalence of polypharmacy prescription among T2DM patients at this tertiary care department and to determine factors that independently predict this polypharmacy prescription. The knowledge of the prevalence of polypharmacy prescription and factors associated with their polypharmacy prescription could be used by Chinese and/or international experts in developing, evaluating and implementing interventions for supporting medicines optimisation (and deprescribing) in Ningbo and beyond in China.

## Materials and methods

### Study design, data source and period

The 5-year study period was from 1 July 2012 to 30 June 2017. An existing computerised medical records database was used in conducting a retrospective cross-sectional study. The database includes information on all patients from their admission to discharge, including their socio-demographic and behavioural information, medical and surgical history, diagnostics (including laboratory results) and prescribed medicines. As this is a medical records database, the medico-nursing team is responsible for data entry on to the database. Another independent team of hospital staff checks the quality of the data and is responsible for the overall management of the database. On our request and with permission from the Research Ethics Committee at the Ningbo First Hospital, China, the data were extracted from the database by a dedicated engineer.

### Study population, inclusion and exclusion criteria

The study included adult (≥18 years of age) T2DM inpatients who were discharged from the tertiary care department for the first time during the study period. If a patient had more than one hospitalisation during the study period, only data relevant to the index event (ie, first hospitalisation) were extracted. Although the database includes information on all inpatients from their admission to discharge, we extracted the prescription data at the time of discharge, as medicines were prescribed as part of their long-term treatment plan. The study excluded those diagnosed with type 1 diabetes, secondary diabetes, gestational diabetes, unknown type of diabetes or endocrine diseases (such as hyperthyroidism and Cushing syndrome).

### Study variables

We extracted the following independent variables from the database: age (18–39 years (younger age), 40–59 years (middle age) or ≥60 years (older age)) [25], sex (male or female), education (university/college, class 7–12, class 1–6 or no qualifications), occupation (manual workers (ie, more physical than mental work), non-manual workers (ie, more mental than physical work) or never worked/retired), marital status (married or single/divorced/widowed), residence (urban or rural based on the “hukou” system (ie, residence registration system in China)) [26], health insurance, smoking (current status), alcohol drinking (current status), duration of T2DM (≤1 year, >1–5 years, >5–10 years or >10 years), blood glucose level (glycated haemoglobin (HbA1c)—<7% (good) or ≥7% (poor)) [27]; estimated using the high-performance liquid chromatographic (HPLC) method, using the D-10 Hemoglobin Analyzer (Bio-Rad, USA)), length of hospital stay (≤5 days, >5–10 days or >10 days) and comorbidities (ie, ≥1 comorbidities and were coded using the International Statistical Classification of Diseases, 10^th^ revision (ICD-10)) [28]. The dependent variable extracted was prescribed medicines at discharge, which were coded using the World Health Organization’s Anatomical Therapeutic and Chemical (ATC) Classification [29]. A polypill was counted as a single medicine. Polypharmacy prescription was defined as the simultaneous prescription of ≥5 medicines by the clinician at the time of discharge for daily usage by the patient as part of his/her long-term treatment plan [17,30,31].

### Ethics

Ethics approval was obtained from the Research Ethics Committee at the Ningbo First Hospital, China. The authors had no access to information that could identify individual participants during data analysis. Therefore, as per the research ethics rules, no informed consent was necessary.

### Statistical analyses

Over a 5-year period, amongst T2DM patients, the prevalence of polypharmacy prescription was calculated. Numbers and proportions were calculated for categorical variables. Means and standard deviations (SDs) were calculated for normally distributed continuous variables. Simple logistic regression method was used to explore the relationship between independent variables and polypharmacy prescription. To find any independent relationship, multiple logistic regression models were developed using backward stepwise regression analyses and we included all the independent variables. We also carried out sensitivity analyses–in multiple logistic regression models, only those independent variables with a p-value of ≤0.20 in simple logistic regressions were included. We calculated Odds ratios (ORs) and their respective 95% confidence intervals (CIs). IBM SPSS Statistics Version 20.0 for Windows was used for data analysis. In addition, we carried out logistic regression analyses to explore the association between neoplasms (no, benign and malignant) and polypharmacy prescription.

## Results

**Fig 1:**
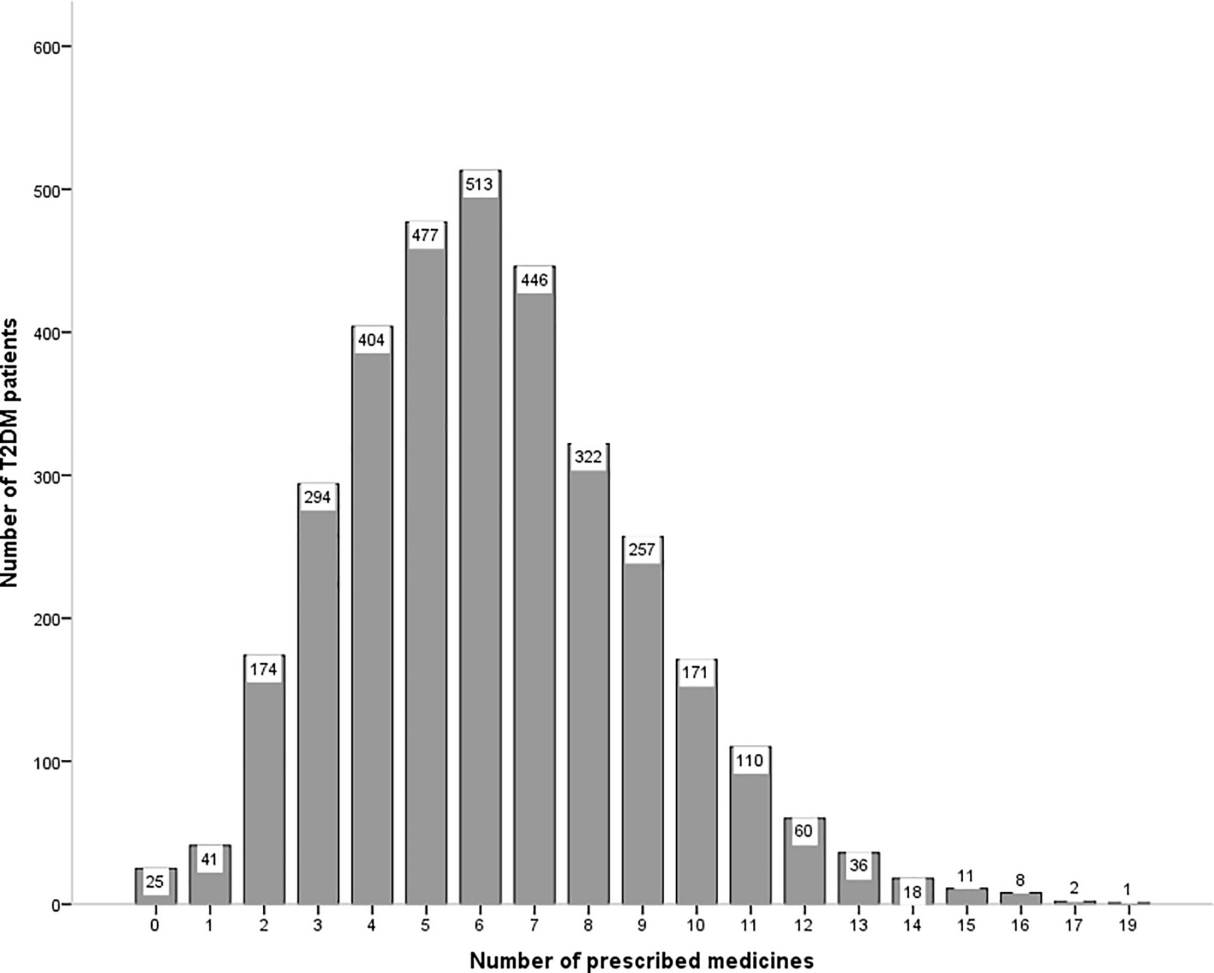
Number of prescribed medicines in our study.

The study inclusion criteria were satisfied by 3370 T2DM patients. The mean (±SD) age of T2DM patients was 62.9 (±13.8) years and around 51% (n = 1713) of them were male. Over a 5-year period, amongst T2DM patients, the prevalence of polypharmacy prescription was 72.2% (n = 2432). Fig 1 shows the number of medicines that were prescribed in our study. Those who were prescribed polypharmacy, the mean (± SD) number of comorbidities and medicines were 5 (±2) and 8 (±2), respectively.

Table 1 shows the most common medicines which were prescribed in our study. 96.7% of T2DM patients were on antidiabetic agents (79.8% were on oral antidiabetic drugs and 74.8% were on insulin), 36.9% were on antithrombotic agents and 69.5% were on lipid modifying agents.

**Table 1:** Most commonly prescribed medicines in our study.

Classification of medicines (based on ATC classification)	Number of T2DM patients n(%)
***Alimentary tract and metabolism (A)***	3312(98.3)
**Drugs used in diabetes (A10)**	3258(96.7)
**Blood glucose lowering drugs, excl. insulins (A10B)**	2688(79.8)
Alpha glucosidase inhibitors (A10BF)	1948(57.8)
Biguanides (A10BA)	1651(49.0)
Repaglinide/nateglinide (A10BX02, A10BX03)	476(14.1)
Sulfonamides (A10BB)	286(8.5)
Thiazolidinediones (A10BG)	71(2.1)
**Insulin and analogues (A10A)**	2520(74.8)
Insulins and analogues for injection, intermediate/long-acting (A10AC, A10AE)	1841(54.6)
Insulins and analogues for injection, fast-acting (A10AB)	1251(37.1)
Insulins and analogues for injection, intermediate- or long-acting combined with fast-acting (A10AD)	672(19.9)
**Drugs for acid related disorders (A02)**	699(20.7)
Proton pump inhibitors (A02BC)	662(19.6)
***Cardiovascular system (C)***	2790(82.8)
**Lipid modifying agents (C10)**	2343(69.5)
HMG CoA reductase inhibitors (C10AA)	2174(64.5)
Atorvastatin and amlodipine (C10BX03)	49(1.5)
**Agents acting on the renin-angiotensin-aldosterone system (C09)**	1640(48.7)
Angiotensin II antagonists and calcium channel blockers (C09DB)	537(15.9)
Angiotensin II antagonists and diuretics (C09DA)	161(4.8)
**Calcium channel blockers (C08)**	483(14.3)
**Beta blocking agents (C07)**	386(11.5)
**Diuretics (C03)**	254(7.5)
**Cardiac therapy (C01)**	245(7.3)
***Blood and blood forming organs (B)***	1789(53.1)
**Antithrombotic agents (B01)**	1244(36.9)
Acetylsalicylic acid (B01AC06)	1030(30.6)
***Nervous system (N)***	385(11.4)
***Musculoskeletal system (M)***	283(8.4)
***Anti-infectives for systemic use(J)***	281(8.3)
***Genitourinary system and sex hormones (G)***	204(6.1)
***Respiratory system (R)***	151(4.5)

Table 2 shows the characteristics of T2DM patients with and without polypharmacy prescription. The polypharmacy prescription was found to be associated with age, education, occupation, residence, health insurance, duration of T2DM (>5 years), blood glucose level, length of hospital stay and commodities like neoplasm, other endocrine, nutritional and metabolic diseases, nervous system diseases, circulatory system diseases, skin and subcutaneous tissue diseases, musculoskeletal system and connective tissue diseases, genitourinary system diseases and during pregnancy, childbirth and the puerperium.

**Table 2:** Characteristics of T2DM patients with and without polypharmacy prescription.

	Total (3370)	Polypharmacy prescription No (938) n(%)	Polypharmacy prescription Yes (2432) n(%)	Unadjusted OR (95% CI)
**Age (in years)**				
18–39	205	141(68.8)	64(31.2)	1
40–59	1043	393(37.7)	650(62.3)	3.64(2.64,5.02)
≥60	2122	404(19.0)	1718(81.0)	9.37(6.84,12.83)
**Sex**				
Male	1713	474(27.7)	1239(72.3)	1
Female	1657	464(28.0)	1193(72.0)	0.98(0.85,1.14)
**Education**				
University/college	352	142(40.3)	210(59.7)	1
Class 7–12	1322	400(30.3)	922(69.7)	1.56(1.22,1.99)
Class 1–6	1144	270(23.6)	874(76.4)	2.19(1.70,2.82)
No qualifications	552	126(22.8)	426(77.2)	2.29(1.71,3.06)
**Occupation**				
Manual workers	727	211(29.0)	516(71.0)	1
Non-manual workers	771	319(41.4)	452(58.6)	0.58(0.47,0.72)
Never worked/retired	1872	408(21.8)	1464(78.2)	1.47(1.21,1.78)
**Marital status**				
Married	2895	822(28.4)	2073(71.6)	1
Single/divorced/widowed	475	116(24.4)	359(75.6)	1.23(0.98,1.54)
**Residence**				
Urban	1988	506(25.5)	1482(74.5)	1
Rural	1382	432(31.3)	950(68.7)	0.75(0.65,0.87)
**Health insurance**				
Yes	2882	749(26.0)	2133(74.0)	1
No	488	189(38.7)	299(61.3)	0.56(0.46,0.68)
**Smoking (current status)**				
No	2713	756(27.9)	1957(72.1)	1
Yes	657	182(27.7)	475(72.3)	1.01(0.83,1.22)
**Alcohol drinking (current status)**				
No	3005	845(28.1)	2160(71.9)	1
Yes	365	93(25.5)	272(74.5)	1.14(0.89,1.47)
**Duration of T2DM (in years)**				
≤1	140	66(47.1)	74(52.9)	1
>1–5	780	346(44.4)	434(55.6)	1.12(0.78,1.61)
>5–10	746	227(30.4)	519(69.6)	2.04(1.41,2.94)
>10	1704	299(17.5)	1405(82.5)	4.19(2.94,5.97)
**Blood glucose level (HbA1c)**				
<7%	603	221(36.7)	382(63.3)	1
≥7%	2678	684(25.5)	1994(74.5)	1.69(1.40,2.03)
Unknown	89	33(37.1)	56(62.9)	0.98(0.62,1.56)
**Length of hospital stay (in days)**				
≤5	374	201(53.7)	173(46.3)	1
>5–10	1815	502(27.7)	1313(72.3)	3.04(2.42,3.82)
>10	1181	235(19.9)	946(80.1)	4.68(3.65,6.00)
**Comorbidities**				
**Infectious and parasitic diseases (A00-B99)**				
No	3048	846(27.8)	2202(72.2)	1
Yes	322	92(28.6)	230(71.4)	0.96(0.75,1.24)
**Neoplasms (C00-D48)**				
No	3157	844(26.7)	2313(73.3)	1
Yes	213	94(44.1)	119(55.9)	0.46(0.35,0.61)
**Blood diseases and certain disorders involving the immune mechanism (D50-D89)**				
No	3271	909(27.8)	2362(72.2)	1
Yes	99	29(29.3)	70(70.7)	0.93(0.60,1.44)
Endocrine, nutritional and metabolic diseases (E00-E90)*				
No	449	220(49.0)	229(51.0)	1
Yes	2921	718(24.6)	2203(75.4)	2.95(2.41,3.61)
**Mental and behavioural disorders (F00-F99)**				
No	3275	919(28.1)	2356(71.9)	1
Yes	95	19(20.0)	76(80.0)	1.56(0.94,2.59)
**Nervous system diseases (G00-G99)**				
No	3159	895(28.3)	2264(71.7)	1
Yes	211	43(20.4)	168(79.6)	1.54(1.10,2.18)
**Circulatory system diseases (I00-I99)**				
No	1210	608(50.2)	602(49.8)	1
Yes	2160	330(15.3)	1830(84.7)	5.60(4.76,6.59)
**Respiratory system diseases (J00-J99)**				
No	2908	824(28.3)	2084(71.7)	1
Yes	462	114(24.7)	348(75.3)	1.21(0.96,1.51)
**Digestive system diseases (K00-K93)**				
No	1800	510(28.3)	1290(71.7)	1
Yes	1570	428(27.3)	1142(72.7)	1.06(0.91,1.23)
**Skin and subcutaneous tissue diseases (L00-L99)**				
No	3214	909(28.3)	2305(71.7)	1
Yes	156	29(18.6)	127(81.4)	1.73(1.15,2.60)
**Musculoskeletal system and connective tissue diseases (M00-M99)**				
No	2639	807(30.6)	1832(69.4)	1
Yes	731	131(17.9)	600(82.1)	2.02(1.64,2.48)
**Genitourinary system diseases (N00-N99)**				
No	2501	719(28.7)	1782(71.3)	1
Yes	869	219(25.2)	650(74.8)	1.20(1.01,1.43)
**Pregnancy, childbirth and the puerperium (O00-O99)**				
No	3336	905(27.1)	2431(72.9)	1
Yes	34	33(97.1)	1(2.9)	0.01(0.00,0.08)

*Excludes T2DM

Table 3 shows the multiple backward stepwise logistic regression analyses–all the independent variables were included. The odds of polypharmacy prescription increased with patients’ age (18–39 years: 1; 40–59 years: OR 1.86, 95% CI 1.28–2.71; and ≥60 years: 2.42, 1.65–3.55), duration of T2DM (≤1 year: 1; >5–10 years: 1.70, 1.10–2.62; and >10 years: 2.55, 1.68–3.89), and length of hospital stay (≤5 days: 1; >5–10 days: 2.43, 1.86–3.17; and >10 days: 2.99, 2.24–3.99), and were higher in those with poor blood glucose level (2.09, 1.67–2.62) and with comorbidities like other endocrine, nutritional and metabolic diseases (2.24, 1.76–2.85), circulatory system diseases (4.35, 3.62–5.23), skin and subcutaneous tissue diseases (1.64, 1.04–2.59), and musculoskeletal system and connective tissue diseases (1.61, 1.27–2.03). The odds of polypharmacy prescription were lower in those with comorbidities like neoplasms (0.51, 0.36–0.70) and during pregnancy, childbirth and the puerperium (0.06, 0.01–0.49).

**Table 3:** Multiple backward stepwise logistic regression analyses to determine factors independently associated with polypharmacy prescription—all the independent variables were included.

	Adjusted OR (95% CI)
***Polypharmacy prescription (Yes)***	
**Age (in years)**	
18–39	1
40–59	1.86(1.28,2.71)
≥60	2.42(1.65,3.55)
**Duration of T2DM (in years)**	
≤1	1
>1–5	1.23(0.81,1.88)
>5–10	1.70(1.10,2.62)
>10	2.55(1.68,3.89)
**Length of hospital stay (in days)**	
≤5	1
>5–10	2.43(1.86,3.17)
>10	2.99(2.24,3.99)
**Blood glucose level (HbA1c)**	
<7%	1
≥7%	2.09(1.67,2.62)
Unknown	1.19(0.69,2.06)
**Neoplasms (C00-D48)**	
No	1
Yes	0.51(0.36,0.70)
Endocrine, nutritional and metabolic diseases (E00-E90)*	
No	1
Yes	2.24(1.76,2.85)
**Circulatory system diseases (I00-I99)**	
No	1
Yes	4.35(3.62,5.23)
**Skin and subcutaneous tissue diseases (L00-L99)**	
No	1
Yes	1.64(1.04,2.59)
**Musculoskeletal system and connective tissue diseases (M00-M99)**	
No	1
Yes	1.61(1.27,2.03)
**Pregnancy, childbirth and the puerperium (O00-O99)**	
No	1
Yes	0.06(0.01,0.49)

*Excludes T2DM

Variables age, sex, education, occupation, marital status, residence, health insurance, smoking, alcohol drinking, duration of T2DM, blood glucose level (HbA1c), length of hospital stay, infectious and parasitic diseases, neoplasms, blood diseases, endocrine diseases, mental and behavioural disorders, nervous system diseases, circulatory system diseases, respiratory system diseases, digestive system diseases, skin diseases, musculoskeletal system diseases, genitourinary system diseases, pregnancy and the puerperium were included.

Table 4 shows the sensitivity analyses (multiple logistic regression models)—independent variables with p≤0.20 in simple logistic regressions were included. We found similar results in the sensitivity analyses.

**Table 4:** Sensitivity analyses (multiple logistic regression models)—independent variables with p≤0.20 in simple logistic regressions were included.

	Adjusted OR (95% CI)
***Polypharmacy prescription (Yes)***	
**Age (in years)**	
18–39	1
40–59	1.85(1.27,2.69)
≥60	2.37(1.62,3.47)
**Duration of T2DM (in years)**	
≤1	1
>1–5	1.22(0.80,1.87)
>5–10	1.68(1.09,2.58)
>10	2.50(1.64,3.80)
**Length of hospital stay (in days)**	
≤5	1
>5–10	2.44(1.87,3.18)
>10	3.00(2.25,4.00)
**Blood glucose level (HbA1c)**	
<7%	1
≥7%	2.10(1.68,2.62)
Unknown	1.18(0.68,2.04)
**Neoplasms (C00-D48)**	
No	1
Yes	0.49(0.35,0.68)
Endocrine, nutritional and metabolic diseases (E00-E90)*	
No	1
Yes	2.24(1.76,2.85)
**Circulatory system diseases (I00-I99)**	
No	1
Yes	4.34(3.61,5.21)
**Skin and subcutaneous tissue diseases (L00-L99)**	
No	1
Yes	1.63(1.03,2.57)
**Musculoskeletal system and connective tissue diseases (M00-M99)**	
No	1
Yes	1.57(1.24,1.97)
**Pregnancy, childbirth and the puerperium (O00-O99)**	
No	1
Yes	0.05(0.01,0.44)

*Excludes T2DM

Variables age, education, occupation, marital status, residence, health insurance, duration of T2DM, blood glucose level (HbA1c), length of hospital stay, neoplasms, endocrine diseases, mental and behavioural disorders, nervous system diseases, circulatory system diseases, respiratory system diseases, skin diseases, musculoskeletal system diseases, genitourinary system diseases, pregnancy and the puerperium were included. (Sex, smoking, alcohol drinking, infectious and parasitic diseases, blood diseases, digestive diseases were excluded.)

## Discussion

In our study, around three fourth of T2DM patients were prescribed polypharmacy. Those who were prescribed polypharmacy, the average number of medicines was eight. Globally, similar studies have been conducted in different diabetes populations and settings, during different time periods, using similar or different case definitions of polypharmacy. The prevalence of polypharmacy (≥5 medicines) among T2DM patients in Italy and Brazil was 57% and 85%, respectively [17,21]. In similar studies conducted among diabetes patients in Brazil and Saudi Arabia, it was 57% and 78%, respectively [16,18]. 48% of patients with diabetes and hypertension in Canada were prescribed ≥9 medicines [19]. The average number of medicines prescribed to T2DM patients in India and Germany was five and 12, respectively [15,20]. A standardised universally accepted definition of polypharmacy is not available. A recently published systematic review reported that approximately half of the published studies defined polypharmacy as administering ≥5 medicines to an individual. They found a huge variation in the definition of polypharmacy used, which ranged from ≥2 medicines to ≥11 medicines [32]. Therefore, in our study, we selected the most commonly used definition of polypharmacy ie, the simultaneous prescription of ≥5 medicines.

In T2DM, the core management strategy is to control blood glucose and prevent or manage vascular complications [27]. Similar to our study, in China, the management of uncontrolled blood glucose levels and T2DM complications (if any) are often the two main reasons for hospitalisation of T2DM patients [33]. During their stay at the hospital, the management strategy can be different and may involve multiple essential medicines for treating complex and severe conditions. We found that around 80% of T2DM patients were prescribed oral antidiabetic drugs and a high proportion (75%) were prescribed insulin. In China, if the first line of treatment (oral antidiabetic drugs) fails to control the blood glucose levels, insulin (premix or basal) is recommended and sometimes in combination with oral antidiabetic drugs [34]. All these increases the complexity of T2DM management and the burden of medicines. Drug-drug interactions can be categorised as pharmacodynamics or pharmacokinetics [35]. The risk of a potential drug interaction increases from 13% for two drugs to 82% for ≥7 drugs [36]. For example, drugs like sulphonylurea and aspirin have a high tendency to bind with plasma proteins [37,38]. When these two drugs are administered simultaneously, aspirin can compete with sulphonylurea. Thereafter, aspirin can bind with plasma proteins, displacing sulphonylurea from plasma proteins [38]. Due to this, the level of sulphonylurea in plasma can increase, which in turn can increase the risk of hypoglycemia, especially among those T2DM patients with controlled (near normal) blood glucose levels [39]. Almost two third of T2DM patients in our study were prescribed β-Hydroxy β-methylglutaryl CoA *(*HMG CoA*)* reductase inhibitors like atorvastatin, lovastatin and simvastatin. One of the severe adverse effects of statins is myopathy. These statins are metabolised by an enzyme, cytochrome P450 3A4 (CYP3A4). Calcium channel blockers like amlodipine are inhibitors of CYP3A4. When these two drugs are administered simultaneously, for example, simvastatin plus amlodipine, amlodipine can increase the level of simvastatin in plasma, which in turn can increase the risk of myopathy [35,40]. Thus, there is an urgent need to develop, evaluate and implement interventions to support medicines optimisation (and deprescribing) among T2DM patients.

The odds of polypharmacy prescription increased with patients’ age in our study, as in studies conducted among diabetes patients in Saudi Arabia and Brazil [16,18]. Even in studies conducted among the general population in Switzerland and Japan, the prevalence of polypharmacy increased with age [41,42]. Usually, older patients have more comorbidities and seek care from multiple healthcare providers [43], which may increase the chance of polypharmacy. Even after we adjusted for some known comorbidities, age was found to be independently associated with polypharmacy prescription. Due to the natural senescence process of liver and kidneys in older patients, the risk of drug accumulation is more serious [44,45]. The adverse effect of a drug can be misinterpreted as a sign or symptom of a new disorder and new drugs may be added to the medication list to manage it, which is known as “prescription cascade”. This further complicates the matter by increasing the risk of unwanted drug interactions between existing and new drugs and resulting in adverse drug events, especially among older patients who are already on multiple drugs [46].

The odds of polypharmacy prescription increased with the duration of T2DM in our study. Similarly, a study conducted among T2DM patients in Italy reported that polypharmacy was associated with ≥5 years duration of T2DM (1.93, 1.38–2.70) [17]. In another study conducted among diabetes patients in Brazil, polypharmacy was found to be associated with ≥10 years duration of T2DM (1.64, 1.36–1.98) [16]. With the progression of T2DM, the function and mass of β-cells gradually decline [47]. In T2DM patients, single antidiabetic drugs like metformin, sulfonylureas or thiazolidinediones cannot control their blood glucose levels for a long period of time and as the disease progresses over time, they will need multiple antidiabetic drugs [47]. In addition, the risk of vascular complications increases with the duration of T2DM [48] and thus, will require multiple medicines as part of the management strategy.

We found that the odds of polypharmacy prescription increased with the length of hospital stay in T2DM patients. Similarly, a study conducted among older patients in India reported the association between longer length of hospital stay and polypharmacy (3.14, 2.09–4.71 for 10 to <15 days length of hospital stay; and 5.74, 2.43–13.51 for ≥15 days length of hospital stay) [49]. Length of hospital stay is a known indicator of the severity of T2DM, its comorbidities and complications [50]. The more complex and severe the case is, the more medicines are required to manage it.

The odds of polypharmacy prescription were higher in those with poor blood glucose level in our study. In T2DM patients with poor blood glucose levels, additional antidiabetic drugs are needed, as is evident from another study conducted among T2DM patients in the USA [51]. It should be noted that poor blood glucose levels could be due to comorbidities (eg, other metabolic disorders), requiring additional antidiabetic drugs. In our study, comorbidities were adjusted for in the models.

We found that the average number of comorbidities was five among those who were prescribed polypharmacy. The odds of polypharmacy prescription were higher in those with comorbidities like other endocrine, nutritional and metabolic diseases (such as obesity, hyperuricaemia, hyperlipidaemia and T2DM micro-vascular complications), circulatory system diseases (such as hypertension and T2DM macro-vascular complications like coronary heart disease and stroke), skin and subcutaneous tissue diseases, and musculoskeletal system and connective tissue diseases. Similarly, in a study conducted among T2DM patients in Ireland, those with one and ≥5 chronic illnesses were prescribed three and eight medicines on an average, respectively [52]. In another study conducted among T2DM in Italy, polypharmacy was found to be associated with comorbidities, assessed using the Cumulative Illness Rating Scale (CIRS) (1.9, 1.41–2.54 for CIRS ≥2) [17]. In a study conducted among diabetes patients in Saudi Arabia, polypharmacy was found to be associated with cardiovascular diseases (2.89, 2.54–3.29), mental health conditions (2.19, 1.74–2.76), respiratory diseases (2.42, 1.92–3.03) and musculoskeletal diseases (3.16, 2.31–4.30) [18]. Usually, T2DM patients will have at least one comorbidity and approximately 40% of them will have ≥3 comorbidities [52,53]. It should be noted that T2DM patients are also prone to skin diseases [54]. As mentioned above, there is a high risk of “prescription cascade” in older patients, and itching is one of the known adverse effects of drugs, which in turn will need medicines [55,56]. One of the prominent clinical symptoms of the musculoskeletal system and connective tissue diseases is the pain, which will also need medicines [57].

In this study, the odds of polypharmacy prescription were lower in those with comorbidities like neoplasms and during pregnancy, childbirth and the puerperium. The benign neoplasms do not require any medicine such as uterine leiomyoma, the pituitary tumour with no function and hepatic haemangioma. We found that the odds of polypharmacy prescription were lower in those with malignant neoplasms compared to no tumours (S1 Table). It should be noted that patients with malignant neoplasms requiring multiple medicines are primarily treated elsewhere (and not in this tertiary care department). Those admitted to this tertiary care department usually have reduced life expectancy, and the primary management aim is to provide palliative care with minimal medicines [27,34]. In fact, their blood glucose, blood pressure and lipid targets are relaxed and thus, multiple medicines are avoided [27,34]. During pregnancy, only insulin is approved for controlling blood glucose levels in China [27,34]. Similarly, many medicines are not prescribed or used in pregnancy to avoid known and unknown adverse effects on the foetus [58].

The study has a number of strengths and weaknesses. As far as we are aware, this was the first study in China to explore polypharmacy prescription among T2DM patients. Our study findings could be taken into consideration in future interventional studies aimed at supporting medicines optimisation (and deprescribing) among these patients. This study was conducted among T2DM patients who were discharged from the tertiary care department and thus, the generalisability of study findings is limited to similar populations and settings. As mentioned before, studies conducted in other diabetes (including T2DM) populations and settings have reported a range of prevalence figures [15–21]. In China, studies conducted in community settings have reported a much lower prevalence of polypharmacy (6–18%) [22–24]. Our study focused on prescribed western medicines. Data on other important components such as over-the-counter medicines, traditional Chinese medicines and actual usage of medicines were not available in the database and needs further exploration to provide a more complete picture of the issue. Missing data, which could lead to bias, were low in this study. A sample with missing values for the variable was included in the regression analyses. This was a retrospective study, which used an existing computerised medical records database. The primary purpose of the development of this database was clinical and not research and thus, data quality issues (such as accuracy and reliability) of routinely collected data cannot be ignored. However, hospitalised patients, due to disease severity, are usually precisely monitored, and this could have improved the data quality. Recall error could have been a problem with self-reported data (eg, duration of T2DM). The inaccurate measurement of the variable could mean that patients were assigned to the wrong group, which resulted in an incorrect estimation of the association between duration of T2DM and polypharmacy prescription. There is a possibility that our study findings were the outcome of other factors (such as cognitive performance, diet and physical activity) not present in the database and so, not adjusted for in the models [16, 59–61]. Similarly, only the current status of smoking and alcohol drinking was available in the database and the past history was missing. Being a cross-sectional study, it was not possible to assess the causal relationship between different variables and polypharmacy prescription. We suggest conducting a longitudinal study among T2DM patients to evaluate the impact of various factors (these as well as other potential factors) on polypharmacy prescription. In addition, ours was a hospital-based study, and a population-based study might provide a different picture of the issue. The reasons could be different population characteristics, including their disease severity and healthcare-seeking behaviour.

In conclusion, around three fourth of T2DM patients at the tertiary care department were prescribed polypharmacy, and the predictors were identified. The study findings could be taken into consideration in future interventional studies aimed at supporting medicines optimisation (and deprescribing) among these patients.

## Supporting information

S1 Table Association between neoplasms and polypharmacy prescription. (DOCX)

S1 Dataset Polypharmacy prescription among type 2 diabetes patients in Ningbo, China. (XLSX)

## References

[1] International Diabetes Federation. IDF diabetes atlas. 8th edition Brussels: International Diabetes Federation; 2017.

[2] Yao DZ, Sun XH, Li JH. Prevalence and risk factors of diabetes in people over 40 years of age in Ningbo city area. Modern Practical Medicine. 2016; 28:1343–1345.

[3] Li J, Chattopadhyay K, Xu M, Chen Y, Hu F, Chu J, et al Glycaemic control in type 2 diabetes patients and its predictors: a retrospective database study at a tertiary care diabetes centre in Ningbo, China. BMJ Open. 2018; 8:e019697 10.1136/bmjopen-2017-019697 29581203 PMC5875602

[4] The Public Health Service Platform of Ningbo. Ningbo First Hospital. Available from: http://gzjk.nbws.gov.cn/f/mech?mechId=121

[5] Ministry of Health of the People’s Republic of China. The measures for the administration of the hospital grade. Beijing: Ministry of Health of the People’s Republic of China; 1989.

[6] Marengoni A, Onder G. Guidelines, polypharmacy, and drug-drug interactions in patients with multimorbidity. BMJ. 2015; 350:h1059 10.1136/bmj.h1059 25761379

[7] Doan J, Zakrzewski-Jakubiak H, Roy J, Turgeon J, Tannenbaum C. Prevalence and risk of potential cytochrome P450-mediated drug-drug interactions in older hospitalized patients with polypharmacy. Ann Pharmacother. 2013; 47:324–332. 10.1345/aph.1R621 23482734

[8] Field TS, Gurwitz JH, Harrold LR, Rothschild J, DeBellis KR, Seger AC, et al Risk factors for adverse drug events among older adults in the ambulatory setting. J Am Geriatr Soc. 2004; 52:1349–1354. 10.1111/j.1532-5415.2004.52367.x 15271125

[9] Marengoni A, Pasina L, Concoreggi C, Martini G, Brognoli F, Nobili A, et al Understanding adverse drug reactions in older adults through drug-drug interactions. Eur J Intern Med. 2014; 25:843–846. 10.1016/j.ejim.2014.10.001 25312593

[10] Grant RW, Devita NG, Singer DE, Meigs JB. Polypharmacy and medication adherence in patients with type 2 diabetes. Diabetes Care. 2003; 26:1408–1412. 10.2337/diacare.26.5.1408 12716797

[11] Montiel-Luque A, Núñez-Montenegro AJ, Martín-Aurioles E, Canca-Sanchez JC, Toro-Toro MC, González-Correa JA, et al Medication-related factors associated with health-related quality of life in patients older than 65 years with polypharmacy. PLoS One. 2017; 12:e0171320 10.1371/journal.pone.0171320 28166266 PMC5293190

[12] Willey CJ, Andrade SE, Cohen J, Fuller JC, Gurwitz JH. Polypharmacy with oral antidiabetic agents: an indicator of poor glycemic control. Am J Manag Care. 2006; 12:435–440. 16886886

[13] Marcum ZA, Amuan ME, Hanlon JT, Aspinall SL, Handler SM, Ruby CM, et al Prevalence of unplanned hospitalizations caused by adverse drug reactions in older veterans. J Am Geriatr Soc. 2012; 60:34–41. 10.1111/j.1532-5415.2011.03772.x 22150441 PMC3258324

[14] García A LM, Villarreal R E, Galicia R L, Martinez G L, Vargas D ER. The cost of polypharmacy in patients with type 2 diabetes mellitus. Rev Med Chil. 2015; 143:606–611. 10.4067/S0034-98872015000500008 26203572

[15] Bauer S, Nauck MA. Polypharmacy in people with type 1 and type 2 diabetes is justified by current guidelines: a comprehensive assessment of drug prescriptions in patients needing inpatient treatment for diabetes-associated problems. Diabet Med. 2014; 31:1078–1085. 10.1111/dme.12497 24824448

[16] da Silva MRR, Diniz LM, Santos JBRD, Reis EA, Mata ARD, Araújo VE, et al Drug utilization and factors associated with polypharmacy in individuals with diabetes mellitus in Minas Gerais, Brazil. Cien Saude Colet. 2018; 23:2565–2574. 10.1590/1413-81232018238.10222016 30137126

[17] Noale M, Veronese N, Cavallo Perin P, Pilotto A, Tiengo A, Crepaldi G, et al Polypharmacy in elderly patients with type 2 diabetes receiving oral antidiabetic treatment. Acta Diabetol. 2016; 53:323–330. 10.1007/s00592-015-0790-4 26155958

[18] Alwhaibi M, Balkhi B, Alhawassi TM, Alkofide H, Alduhaim N, Alabdulali R, et al Polypharmacy among patients with diabetes: a cross-sectional retrospective study in a tertiary hospital in Saudi Arabia. BMJ Open. 2018; 8:e020852 10.1136/bmjopen-2017-020852 29794097 PMC5988096

[19] McCracken R, McCormack J, McGregor MJ, Wong ST, Garrison S. Associations between polypharmacy and treatment intensity for hypertension and diabetes: a cross-sectional study of nursing home patients in British Columbia, Canada. BMJ Open. 2017; 7:e017430 10.1136/bmjopen-2017-017430 28801438 PMC5724061

[20] Indu R, Adhikari A, Maisnam I, Basak P, Sur TK, Das AK. Polypharmacy and comorbidity status in the treatment of type 2 diabetic patients attending a tertiary care hospital: an observational and questionnaire-based study. Perspect Clin Res. 2018; 9:139–144. 10.4103/picr.PICR_81_17 30090713 PMC6058506

[21] da Silva Córralo V, Marconatto Binotto V, Bohnen LC, Gonzaga Dos Santos GA, De-Sá CA. Polypharmacy and associated factors in elderly diabetic. Rev Salud Publica (Bogota). 2018; 20:366–372. 30844011 10.15446/rsap.V20n3.50304

[22] Yang M, Lu J, Hao Q, Luo L, Dong B. Does residing in urban or rural areas affect the incidence of polypharmacy among older adults in western China? Arch Gerontol Geriatr. 2015; 60:328–333. 10.1016/j.archger.2014.11.004 25440757

[23] Dong L, Yan H, Wang D. Polypharmacy and its correlates in village health clinics across 10 provinces of Western China. J Epidemiol Community Health. 2010; 64:549–553. 10.1136/jech.2008.085415 19854749

[24] Lu J, Yang M, Luo L, Hao Q, Dong B. Polypharmacy among nonagenarians/centenarians in rural China. Intern Med J. 2014; 44:1193–1199. 10.1111/imj.12534 25039536

[25] Han C, Zhang M, Luo X, Wang C, Yin L, Pang C, et al Secular trends in the prevalence of type 2 diabetes in adults in China from 1995 to 2014: a meta-analysis. J Diabetes. 2017; 9:450–461. 10.1111/1753-0407.12440 27282985

[26] The National People’s Congress of the People’s Republic of China. Regulations of the People’s Republic of China on residence registration. Available from: http://www.npc.gov.cn/wxzl/gongbao/2000-12/10/content_5004332.htm

[27] Chinese Diabetes Society. Chinese guideline for type 2 diabetes (2013 ed). Chin J Endocrinol Metab. 2014; 30:893–942.

[28] International Statistical Classification of Diseases, 10th revision Geneva, Switzerland: World Health Organization; 2005.

[29] WHO Collaborating Centre for Drug Statistics Methodology. ATC/DDD Index 2018. Available from: https://www.whocc.no/atc_ddd_index/

[30] Blanco-Reina E, Ariza-Zafra G, Ocaña-Riola R, León-Ortíz M, Bellido-Estévez I. Optimizing elderly pharmacotherapy: polypharmacy vs. undertreatment. Are these two concepts related? Eur J Clin Pharmacol. 2015; 71:199–207. 10.1007/s00228-014-1780-0 25380629

[31] Gnjidic D, Hilmer SN, Blyth FM, Naganathan V, Waite L, Seibel MJ, et al Polypharmacy cutoff and outcomes: five or more medicines were used to identify community-dwelling older men at risk of different adverse outcomes. J Clin Epidemiol. 2012; 65:989–995. 10.1016/j.jclinepi.2012.02.018 22742913

[32] Masnoon N, Shakib S, Kalisch-Ellett L, Caughey GE. What is polypharmacy? A systematic review of definitions. BMC Geriatr. 2017; 17:230 10.1186/s12877-017-0621-2 29017448 PMC5635569

[33] Lin W, Chen C, Guan H, Du X, Li J. Hospitalization of elderly diabetic patients: characteristics, reasons for admission, and gender differences. BMC Geriatr. 2016; 16:160 10.1186/s12877-016-0333-z 27595573 PMC5011894

[34] Chinese Diabetes Society. Chinese guideline for type 2 diabetes (2017 ed). Chin J Diabetes Mellitus. 2018; 10:4–67.

[35] Triplitt Curtis. Drug interactions of medications commonly used in diabetes. Diabetes Spectrum. 2006; 19: 202–211.

[36] Goldberg RM, Mabee J, Chan L, Wong S. Drug-drug and drug-disease interactions in the ED: analysis of a high-risk population. Am J Emerg Med. 1996; 14:447–450. 10.1016/S0735-6757(96)90147-3 8765105

[37] White JR, Campbell RK. Dangerous and common drug interactions in patients with diabetes mellitus. Endocrinol Metab Clin North Am. 2000; 29:789–802. 11149162 10.1016/s0889-8529(05)70164-x

[38] Kubacka RT, Antal EJ, Juhl RP, Welshman IR. Effects of aspirin and ibuprofen on the pharmacokinetics and pharmacodynamics of glyburide in healthy subjects. Ann Pharmacother. 1996; 30:20–6. 10.1177/106002809603000103 8773160

[39] Dongwei Yu, Qinhua Gu. Hypoglycemia and coma associated with gliclazide and glipizide. Adverse Drug Reactions Journal. 2011; 13:114–115.

[40] Hirota T, Ieiri I. Drug-drug interactions that interfere with statin metabolism. Expert Opin Drug Metab Toxicol. 2015; 11:1435–1447. 10.1517/17425255.2015.1056149 26058399

[41] Abolhassani N, Castioni J, Marques-Vidal P, Vollenweider P, Waeber G. Determinants of change in polypharmacy status in Switzerland: the population-based CoLaus study. Eur J Clin Pharmacol. 2017; 73:1187–1194. 10.1007/s00228-017-2288-1 28634642

[42] Onoue H, Koyama T, Zamami Y, Hagiya H, Tatebe Y, Mikami N, et al Trends in polypharmacy in Japan: a nationwide retrospective study. J Am Geriatr Soc. 2018; 66:2267–2273. 10.1111/jgs.15569 30291747

[43] Gibson TB, Ozminkowski RJ, Goetzel RZ. The effects of prescription drug cost sharing: a review of the evidence. Am J Manag Care. 2005; 11:730–740. 16268755

[44] Mühlberg W, Platt D. Age-dependent changes of the kidneys: pharmacological implications. Gerontology. 1999; 45:243–253. 10.1159/000022097 10460985

[45] Klotz U. Pharmacokinetics and drug metabolism in the elderly. Drug Metab Rev. 2009; 41:67–76. 10.1080/03602530902722679 19514965

[46] Rochon PA, Gurwitz JH. The prescribing cascade revisited. Lancet. 2017; 389:1778–1780. 10.1016/S0140-6736(17)31188-1 28495154

[47] Kahn SE, Haffner SM, Heise MA, Herman WH, Holman RR, Jones NP, et al Glycemic durability of rosiglitazone, metformin, or glyburide monotherapy. N Engl J Med. 2006; 355:2427–2443. 10.1056/NEJMoa066224 17145742

[48] Zoungas S, Woodward M, Li Q, Cooper ME, Hamet P, Harrap S, et al Impact of age, age at diagnosis and duration of diabetes on the risk of macrovascular and microvascular complications and death in type 2 diabetes. Diabetologia. 2014; 57:2465–2474. 10.1007/s00125-014-3369-7 25226881

[49] Harugeri A, Joseph J, Parthasarathi G, Ramesh M, Guido S. Prescribing patterns and predictors of high-level polypharmacy in the elderly population: a prospective surveillance study from two teaching hospitals in India. Am J Geriatr Pharmacother. 2010; 8:271–280. 10.1016/j.amjopharm.2010.06.004 20624616

[50] Huang DJ, Xie LZ, Qiu Y. Analysis of factors affecting the length of hospital stay for patients with diabetes. Exp Clin Endocrinol Diabetes. 2016; 124:5–10. 10.1055/s-0035-1565059 26588492

[51] Willey CJ, Andrade SE, Cohen J, Fuller JC, Gurwitz JH. Polypharmacy with oral antidiabetic agents: an indicator of poor glycemic control. Am J Manag Care. 2006; 12:435–440. 16886886

[52] Teljeur C, Smith SM, Paul G, Kelly A, O'Dowd T. Multimorbidity in a cohort of patients with type 2 diabetes. Eur J Gen Pract. 2013; 19:17–22. 10.3109/13814788.2012.714768 23432037

[53] Wolff JL, Starfield B, Anderson G. Prevalence, expenditures, and complications of multiple chronic conditions in the elderly. Arch Intern Med. 2002; 162:2269–2276. 12418941 10.1001/archinte.162.20.2269

[54] Makrantonaki E, Jiang D, Hossini AM, Nikolakis G, Wlaschek M, Scharffetter-Kochanek K, et al Diabetes mellitus and the skin. Rev Endocr Metab Disord. 2016;17:269–282. 10.1007/s11154-016-9373-0 27432328

[55] Valdes-Rodriguez R, Stull C, Yosipovitch G. Chronic pruritus in the elderly: pathophysiology, diagnosis and management. Drugs Aging. 2015; 32:201–215. 10.1007/s40266-015-0246-0 25693757

[56] Leslie TA. Itch management in the elderly. Curr Probl Dermatol. 2016; 50:192–201. 10.1159/000446094 27578088

[57] Carmona-Torres JM, Cobo-Cuenca AI, Recio-Andrade B, Laredo-Aguilera JA, Martins MM, Rodriguez-Borrego MA, et al Prevalence and factors associated with polypharmacy in the older people: 2006–2014. J Clin Nurs. 2018; 27:2942–2952. 10.1111/jocn.14371 29603814

[58] Ito S. Mother and child: medication use in pregnancy and lactation. Clin Pharmacol Ther. 2016; 100:8–11. 10.1002/cpt.383 27272612

[59] Alzner R, Bauer U, Pitzer S, Schreier MM, Osterbrink J, Iglseder B, et al Polypharmacy, potentially inappropriate medication and cognitive status in Austrian nursing home residents: results from the OSiA study. Wien Med Wochenschr. 2016; 166:161–165. 10.1007/s10354-015-0428-8 26847440

[60] Jyrkkä J, Enlund H, Lavikainen P, Sulkava R, Hartikainen S. Association of polypharmacy with nutritional status, functional ability and cognitive capacity over a three-year period in an elderly population. Pharmacoepidemiol Drug Saf. 2011; 20:514–522. 10.1002/pds.2116 21308855

[61] Little MO. Updates in nutrition and polypharmacy. Curr Opin Clin Nutr Metab Care. 2018; 21:4–9. 10.1097/MCO.0000000000000425 29016367

