# Supplementary material for: Prevalence and predictors of polypharmacy prescription among type 2 diabetes patients at a tertiary care department in Ningbo, China: A retrospective database study
Source: PLoS One. 2019 Jul 17;14(7):e0220047. doi: 10.1371/journal.pone.0220047 (PMC6636754; doi:10.1371/journal.pone.0220047)
Supplement: S1 Table — (DOCX) [file pone.0220047.s001.docx]

S1 Table. Association between neoplasms and polypharmacy prescription.

|  | **Total**  **(3370)** | **Polypharmacy**  **prescription**  **No (938)**  **n(%)** | **Polypharmacy**  **prescription**  **Yes (2432)**  **n(%)** | **Unadjusted OR (95% CI)** | **Adjusted^**  **OR (95% CI)** |
| --- | --- | --- | --- | --- | --- |
| **Neoplasms (C00-D48)** |  |  |  |  |  |
| No | 3157 | 844(26.7) | 2313(73.3) | 1 | 1 |
| Benign | 152 | 57(37.5) | 95(62.5) | 0.61(0.43,0.85) | 0.69(0.47,1.03) |
| Malignant | 61 | 37(60.7) | 24(39.3) | 0.24(0.14,0.40) | 0.21(0.12,0.38) |

^Adjusted for only those variables with p≤0.20 in simple logistic regressions (sensitivity analysis).
